# What is the best way to evaluate social prescribing? A qualitative feasibility assessment for a national impact evaluation study in England

**DOI:** 10.1177/13558196231212854

**Published:** 2023-12-15

**Authors:** Abimbola Ayorinde, Amy Grove, Iman Ghosh, Jenny Harlock, Edward Meehan, Natalie Tyldesley-Marshall, Adam Briggs, Aileen Clarke, Lena Al-Khudairy

**Affiliations:** 1 Division of Health Sciences, 2707 University of Warwick, Coventry, UK; 2 2541 Monash University, Melbourne, VIC, Australia

**Keywords:** social prescribing, link worker, feasibility, service evaluation, qualitative

## Abstract

**Objectives:**

Despite significant investment in social prescribing in England over the last decade, we still do not know if it works, or how models of social prescribing fit within wider health and care policy and practice. This study explores current service delivery structures and assesses the feasibility of a national evaluation of the link worker model.

**Methods:**

Semi-structured interviews were conducted between May and September 2020, with 25 key informants from across social prescribing services in England. Participants included link workers, voluntary, community and social enterprise staff, and those involved in policy and decision-making for social prescribing services. Interview and workshop transcripts were analysed thematically, adopting a framework approach.

**Results:**

We found differences in how services are provided, including by individual link workers, and between organisations and regions. Standards, referral pathways, reporting, and monitoring structures differ or are lacking in voluntary services as compared to clinical services. People can self-refer to a link worker or be referred by a third party, but the lack of standardised processes generated confusion in both public and professional perceptions of the link worker model. We identified challenges in determining the appropriate outcomes and outcome measures needed to assess the impact of the link worker model.

**Conclusions:**

The current varied service delivery structures in England poses major challenges for a national impact evaluation. Any future rigorous evaluation needs to be underpinned with national standardised outcomes and process measures which promote uniform data collection.

## Introduction

Social prescribing involves referring people to non-medical, community, or social activity. It is offered to those with long-term health conditions, poor mental health, and complex social needs.^1^ Social prescribing was developed in the United Kingdom (UK) and has been adopted by many countries globally, although specific approaches vary between and within countries.^2^ In England, the Department of Health and Social Care has endorsed social prescribing as an initiative in primary care to function alongside general practitioner (GP) services to offer community-based support.^3^ The policy target is that 900,000 people will be referred into social prescribing services by 2023/24,^3^ potentially reducing the demands on primary care providers.

Despite significant investments and the national roll out of social prescribing services over the last decade, studies have failed to provide sufficient evidence to establish the effectiveness and or cost-effectiveness of social prescribing in England,^4^ or how models of social prescribing fit within wider health and care policy and practice.

### The link worker model of social prescribing

The link worker model is the model of social prescribing endorsed by the National Health Services (NHS).^3^ In 2018, the UK government announced the allocation of £891 million to English Primary Care Networks (PCNs) to employ additional staff, including social prescribing link workers.^5^ The role of link workers is to support individuals to access appropriate activities to meet their needs. Since social prescribing often involves a series of interventions, rather than just a single one, link workers can potentially contribute to successful uptake of social prescribing.^6^

The premise of the link worker model is that dedicated link workers can spend more time with people. They can help to develop a personalised plan to help improve a patient’s health and wellbeing.^6^ The link worker model also supports more formalised access to voluntary, community, and social enterprise (VCSE) organisations, and can even support people to establish new community groups themselves.^7^ However, members of charitable organisations have raised concerns about the lack of the investment needed to match increased service demand generated by link worker referrals.^7^

The link worker model of social prescribing is a complex intervention particularly due to differences in how it is implemented within and between settings, the complexity of people’s needs, its personalised approach, different referral routes, and lack of specific outcomes.^4^ Although the model has been implemented across various countries,^2^ evaluation for the different groups of people this intervention serves has not been undertaken.^4^ We were commissioned by the National Institute for Health and Social Care Research Health and Social Care Delivery Research Programme to explore the feasibility of evaluating the impact of the social prescribing link worker model. We set out to answer the following two questions:

• What is the best way to evaluate social prescribing?
• Is it possible to determine the impact of social prescribing?

Our aim was to explore current service delivery of the link worker model and to understand the potential challenges and enablers which may influence a future impact evaluation.

This manuscript was completed in accordance with the consolidated criteria for reporting qualitative research.^8^

## Ethical approval

Ethical approval was obtained from the University of Warwick Biomedical and Scientific Research Ethics Committee (Reference number BSREC 93/19-20). The study protocol is publicly available.^9^

## Methods

### Participants and recruitment

Semi-structured interviews with those who deliver social prescribing services were undertaken between May and September 2020 using a prespecified interview guide (see online supplement Table S1).

We did not interview users of link worker services. This was because it was outside the scope of our work. This was a commissioned piece of work, and the funder was specifically interested in technical and scientific issues relating to an evaluation design, focussing on the service provision aspect.

We identified potential interview participants through contacts in the NHS England (NHSE) social prescribing network. We invited national and regional leads of the Social Prescribing Network (including in the East of England, London, West Midlands, Yorkshire and Humber, North-West, South-East, South-West, and North-East). We invited those leads to identify potential participants in sites delivering services with both established and newly developed link worker services. We also invited representatives from onward referral organisations – for example, organisations commissioned by local authorities, NHS, or VCSE organisations.

Members of the research team (LA-K, JH, and IG) contacted potential participants via email to invite them to participate. A description of the study’s aims and objectives was provided to each potential participant. We aimed for a maximum variation sample in terms of geographical region of England, time spent working in social prescribing (months/years), and level of seniority. We used a snowballing strategy, asking interviewed participants to suggest subsequent participants to invite to interview until we had achieved maximal variation in our sample.

We contacted 38 people, and 25 individuals agreed to be interviewed. Those who did not agree either did not respond to our request or were unable to commit the time to take part.

### Data collection and analysis

The interview topic guide was drawn from the questions posed in our commissioning brief and protocol^8^ and the findings of a rapid systematic review of evidence we conducted prior to this primary study.^10^ The guide was reviewed for content and coherence by the project advisory team, (including topic experts, methodologists, a public member, and members of NHSE). As the interviews took place during the COVID-19 pandemic, we added an additional question on the impact of the pandemic on link worker service provision. The interview guide was tested in a pilot interview prior to starting data collection.

Most interviews were conducted via Microsoft Teams (Microsoft Corporation, Redmond, WA, USA), except for three which were conducted via telephone upon interviewee request.

Only researchers and participants were present at the interviews. Each interview was conducted remotely by one member of the research team (LA, IG, JH, and EM), except for four where there were two members present – one member conducted the interview and the other member observed (with participants’ consent) for context, as this other member was participating in the analysis and accuracy checking of transcripts.

Interviewers are qualified mixed-methods researchers holding post-graduate degree(s). At the time of the study (year 2020), JH had more than a decade qualitative experience and 7 years mixed-methods experience, LA had 8 years of experience in mixed-methods research, IG had 1 year of experience in mixed methods and EM had 6 months training in mixed methods as part of post-graduate study and shadowing colleagues.

None of the interviewers had an established relationship with participants before the study commenced. Verbal informed consent was taken at the beginning of each interview and interviews were then recorded. Interviews lasted from 31 min to 147 min in duration.

### Data analysis

Audio-recording was transcribed by a member of the team (IG), checked for accuracy against recordings by another team member (AA), and then imported into NVivo 12 software (QSR International, Warrington, UK)^11^ for storage and analysis. Transcripts were read thoroughly to gain an in-depth understanding of participant responses (AA).

Raw data in transcripts was analysed thematically, using a framework approach.^12,13^ The interview guide (see online supplement Table S1) informed the initial framework which was presented to the larger research team for discussion and feedback. Three members of the team applied the framework to the entire data set (AA, EM, and IG). We then charted the data using Microsoft Excel 365 and searched for patterns in the participant responses with a row for each interview and a column for each code. We searched for similarities and differences in the participant responses and identified any deviant cases. Through iteration, discussion, and independent checking, we identified four major themes summarising the data.

### Coherence checking across the wider social prescribing community

Two national virtual workshops were conducted across the wider social prescribing community to obtain feedback on our preliminary findings, to ensure the face validity of initial findings and to assess practicality. In the first workshop, people with lived experience of social prescribing, people involved in delivering these services and NHSE members attended. This group gave us their views on (1) what the most important evaluation questions to ask were and (2) the impact a formal evaluation of services would have on their community.

The second virtual workshop comprised those people who took part in the interviews, researchers, and NHSE members. We presented our interim findings (as aggregate data) to enable member checking. We sought feedback on the initial findings, cross-checked our interpretations, and discussed gaps which could be areas for future exploration or evaluation.

## Results

### Participants

Of the 25 participants, six were leads (senior staff involved in policy and decision-making for social prescribing services), eight were link workers, and 11 were stakeholders (staff in the NHSE and/or the voluntary, community, and social enterprise sector). Further participant characteristics are presented in online supplement Table S2.

In the quotes below, we identify each participant by their role, the number of years’ experience they have in social prescribing, and the geographical part of England in which they operate. To preserve the anonymity of our participants, we have not included their specific job titles or organisational affiliations.

### Thematic descriptions

The findings of our study are presented via thematic descriptions of the final four themes generated via the framework analysis: (1) communication and the client journey, (2) capturing metrics and outcomes, (3) enablers and challenges to development and implementation of link worker services, and (4) the challenges to impact evaluation. In the text, we support the thematic descriptions with illustrative verbatim participant quotes. We have included additional illustrative quotes in online supplement Table S3.

#### Communication and the client journey

**Figure 1.**
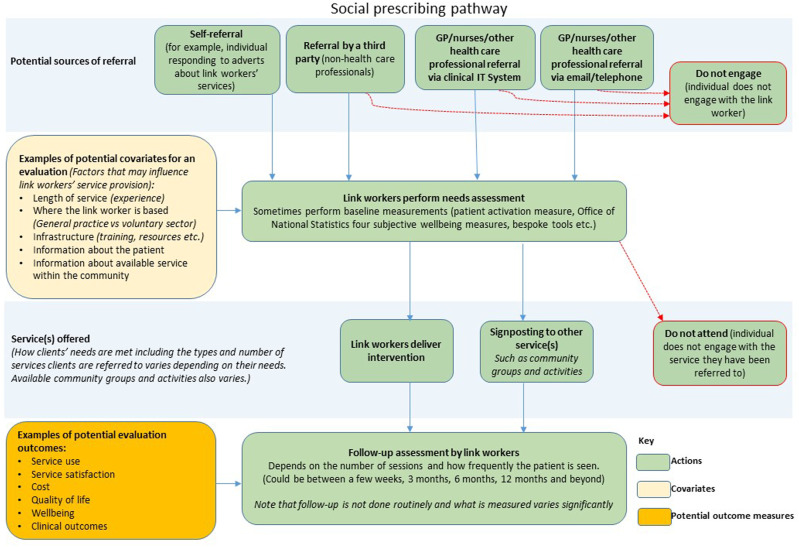
Schematic representation of the link worker model.

There are various routes a client can take on their journey via the link worker model of social prescribing, according to our respondents. Figure 1 presents a schematic representation of these various journeys.

Primarily, however, GPs or nurses introduce the concept of social prescribing to people during a consultation and obtained their consent to refer to link workers. The referral can be made via a multitude of routes including clinical IT platforms hardcopy, softcopy or telephone, or self-referrals. Once a referral is received, the link worker contacts the person, obtains a full assessment of their needs, and completes baseline assessments.

Participants reported that link workers’ onward referrals are to both clinical and non-clinical services. These include support for social isolation, financial or housing advice and encouragement of physical activity, healthier living, and weight management. Other services included bereavement counselling group therapy and support groups for carers.

Many link workers provide their own intervention and do not always refer clients onwards. For example, if the link worker can meet the client’s needs (e.g. with help to complete housing forms).

When asked who accesses services, some participants suggested that the demographic of clients reflected the sociodemographic characteristics of their region. As a senior manager said: ‘It's fairly representative of the local population’ (Stakeholder, 7 years, West Midlands).

Some participants who delivered services suggested clients are more likely to be female, have long-term health and mental conditions, and are more likely to be unemployed. Others suggested that clients were often from more deprived areas, elderly, and were vulnerable people. One link worker said:

The majority of our clients are older, much older – 85 plus. The majority of them live alone. They might have a long-term health condition ... Overall, whatever age, they tend to be more deprived than the general population. (Link worker, 1.5 years, South-East)

One participant noted that living alone is an important predictor as to whether a person would see a link worker:

Living alone was a risk factor for seeing the link worker, and older people were more likely to be referred. So, elderly living alone was the most referred group.

(Lead, 20 years, South-West)

Participants reported changes in service use due to the COVID-19 pandemic. This was because there were few face-to-face services available, reduced resources, and increasing social isolation.

Communication between the many services involved in social prescribing was generally weak. Participants reported no centralised system or standard local process to collate available service information for onward referrals, such as who a service accepts or what actual services they provide. Some link workers created their own personal databases of available services. Others used directories from local authorities, but these were not necessarily shared.

We found instances of strong communication across organisational boundaries when safety concerns were raised – for example, if a person was at risk of suicide. However, client data (such as health and wellbeing data) were not usually communicated to GP practices.

The open-ended nature of link worker services means that establishing an agreed period of intervention and follow-up can be challenging. The nature and the length of follow-up was described as depending on client needs, as well as the link workers’ approach to needs assessment and goals assessment. As described by one participant:

We can move on with that person long term - we don't have a time limit, in terms of how long we support somebody. So we don't necessarily see a cut-off point. (Stakeholder, 2 years, North-East)

We found variations across the country as the services the link workers offered. At one end of the spectrum, we found link workers providing a single intervention that had a hard end-point. An example would be a link worker referring a client to a single activity, such as a weight management programme. At the other end, link workers developed long-term relationships with clients. An example would be where a link worker is continuously engaged with a client to provide support for various needs.

#### Capturing metrics and outcomes

The collection and assessment of outcome data or service provision metrics across the country was sporadic. VCSEs are not routinely required to feed information back to link workers or to GP practices. Nevertheless, some organisations did provide data to such people on attendance, referrals made, and referrals accepted or declined.

In places where outcome data are collected, we found a wide range of metrics captured by link workers (listed in Table 1). However, within that data collection, there is large inconsistency in both what is collected, measurement tools, and the frequency of data collection. Each local area appeared to have their own set of minimum data requirements. However, in some areas, we identified services that did not collect or report on any outcome data.

**Table 1.** Range of data collected by link workers on patients.

• Basic details (name, age, gender, contact details, marital status, religion)
• Whether they have a carer
• Whether they live alone
• Employment status
• Long-term condition
• Disability
• GP practice they are registered with
• Contact with GP practices (to see if consultation rates have been affected)
• Reason for referral (includes various categories such as loneliness, social isolation, emotional wellbeing, being a carer, for exercise, to improve social skills, smoking, and drug/alcohol problems, fire safety advice, welfare benefits advice)
• Attendance/non-attendance
• Number of contacts made
• Number/type of goals
• Number/type of sign postings
• How long people are in the service
• Time spent with person/client
• Outcome measures, including:
- Office of national statistics four subjective wellbeing questions^24^
- Warwick-Edinburgh Mental Wellbeing Scale^25^
- Short Warwick-Edinburgh Mental Wellbeing Scale^26^
- Patient activation measure^27^
- University of California, Los Angeles, loneliness scale^28^
- Wellbeing star^29^
- Measure yourself concerns and wellbeing^30^

Where outcome data are collected, there was a focus on health, wellbeing, and lifestyle outcomes. Feedback from participants suggests, however, that the instruments used are not applicable to their client journeys. For example, the Patient Activation Measure targets people with long-term conditions and is not applicable to use on a person who is referred to a link worker for problems of social isolation. We found that services have attempted to overcome this challenge by developing their own local bespoke data collection and assessment tools. However, it was not clear from our data whether these bespoke tools are validated or comparable to others.

Participants reported that social prescribing is person-led. Therefore, there could be discrepancies in what GPs refer the client for and what the client decides to focus on during their consultation and goal setting appointment with the link worker. This mismatch may lead to conflict in reporting outcomes. Link workers pointed out that they do not collect health information – such as Body Mass Index scores or HbA1c – because medical indicators and health outcomes are not the focus of social prescribing. As one participant said: ‘It’s not a health relationship. Although it’s impacting on health, it’s about being person-centred’ (Stakeholder, 10 months, North-West).

Some of the health data is captured by services the client is referred on to – for example, Body Mass Index was recorded by weight management programmes. Link workers were likely to collate their own non-attendance data as this is part of their service contract, but this information is not needed for onward referrals, which suggests that feedback and audit cycles may not be possible. Participants from the voluntary sector stated that they recorded attendance data regularly, but that systematic collection of non-attendance data was mainly ad hoc.

Many participants (especially those based in NHS GP practices) recorded client information on clinical IT software. These systems allowed data sharing between the link workers and other health care professionals located in a general practice. It was mutually beneficial, as link workers stated that access to internal systems exposes GPs to what link workers are doing and, and therefore, GPs can learn more about the role of a link worker.

Some participants reported using Systemized Nomenclature of Medicine codes in their work. This is a standard clinical terminology used in electronic health records within the NHS and would allow for a national comparison if recorded accurately.^14^ During the pandemic, link workers completed data collection remotely, which created challenges for some clients completing and returning forms. We found examples of link workers using a new computing system developed for link workers called the Elemental system,^15^ and others who had developed their own local systems and databases. There was no consistency or interface between these systems and other NHS IT systems.

#### Enablers and challenges to development and implementation of link worker services

Service engagement was the main challenge to developing and implementing the link working model of social prescribing. Participants stated that many health care professionals, including GPs, and potential clients do not understand the role of the link worker and lack interest in the service. As one link worker said: ‘GPs just aren't particularly interested in social prescribing unless the link workers are based in the surgery running clinics and actually interacting with GP colleagues’ (Link worker, 1.5 years, South-East).

We found a lack of definition of the role of link workers in relation to other health and social care staff (such as social workers). Link workers reported role boundary problems and difficulties encouraging other professional groups to understand their role. This was emphasised in GP practices where Practice Managers did not appear to understand link worker caseload management and, therefore, link workers reported lack of support from practices to do their job effectively. Participants reported excessively high caseloads and a perceived need for increased numbers of link workers to cope with service demand. Link workers also reported perceptions of hostility from staff working in the charity sector because of the blurred boundaries between professional roles. As one link worker recalled:

The charity become anxious the link worker may take some of their jobs. Those charities that provides one-to-one support become hostile and concerned that you are here to do their job. (Link worker, 4 months, North-East)

Coordination of link workers across a region and the financial demand of link worker services on the voluntary sector were reported as problematic. Participants stated that VCSE funding is time- or amount-limited, and therefore, the sustainability on onward referral services is often unknown. One VCSE participant stated that funding from the local authority can be received for ‘up to 2 years’, but others reported a constant ‘struggle to obtain funding’ to stay active. The lack of sustained funding limits the work of those who provide social prescription. As one participant explained: ‘The biggest problems are VCSEs are underfunded in years and that is starting to become an issue’ (Lead, at least 6 years, the East of England).

Our results suggest that funding for link workers does not match that needed to deliver the services. Often, direct funding (from NHSE and local authorities) only supports employment costs (i.e. salary) and other part of the service are either not costed or under-costed. Participants raised concerns about lack of financial support for overheads, equipment (some link workers did not have access to a computer or telephone), adequate management provision and training. There was a reported lack of adequate continual professional development for link workers and link workers mainly work on their own. This can be challenging, as this participant described:

The model that the NHS adopted was to assign one link worker per primary care network. It means that link workers can be very isolated. They have no contact with other link workers, they're not able to share practice and learn from others. And they have no peers to talk to … [Link workers feel] not supported or not having access to the support that they need. (Lead, 20 years, London)

Nevertheless, some participants described virtual team meetings and online forums where link workers can talk to others and receive peer support. These were described as helpful. Connecting into clinical IT platforms appeared to be an important enabler to increasing a sense of connection to the wider service.

#### Challenges to an evaluation of the link worker model

The greatest challenge to an evaluation of the link worker model is the heterogeneity in service provision. As one participant said: ‘It's all developed locally and it's all slightly different locally’ (Stakeholder, 9 years, Yorkshire and Humber).

The inconsistency in outcome assessment tools used is reflected in the lack of national benchmarks for link worker services. Participants highlighted the difficultly in determining the impact and effect of their work, especially at an individual level. A service lead stated:

People talk about reduction in hospital visits, reduction in GP appointments. But I think it's really hard to determine cause and effect - if somebody sees their GP a lot and then suddenly doesn't … Do you attribute that to a win for social prescribing? (Lead, 2 years, North-West)

Participants stated that a client’s experience of the service may be influenced by the personal relationship with the link workers, rather than the quality of the service, hence, it might not be ideal to rely on self-reports alone. Another participant suggested that socioeconomic outcomes, such as return to work, might be a useful outcome measure.

Our participants stated that because link workers work on clients’ presenting problems that could include a number of different health and social care problems, the ‘prescription’ for intervention or onward referral (e.g. of voluntary services) needs to be individualised. This causes problems for consistent assessment and evaluation. This participant describes the conundrum:

[The] underlying root of [the] problem is, how do you measure something that is different for each person? … How should you measure something that is quite nebulous? (Stakeholder, 5 years, North-East)

Participants called for an assessment of how many people are engaging with the link worker model of social prescribing, and whether this could be linked to time saved in GP consultations. However, one participant stated that link workers might *increase* GP usage, through identifying health concerns that need follow up, and that this should be seen as positive. Untangling the direction of impact appears difficult:

Individuals may for a time see their GP more, once social issues are under control … because they actually decided they do want to try and manage their diabetes, rather than just ignoring it. And so that's a positive, but it might look like a negative. (Lead, at least 6 years, the East of England)

## Discussion

The aim of this study was to explore the feasibility of evaluating the impact of the link worker model of social prescribing. We identified several prerequisites needed before an evaluation can be conducted. These include establishing clear and consistent job roles, building robust data systems, routinely collecting data, and ensuring sustained provision of onward referral organisations.

These prerequisites may be impossible to implement in social prescribing, which is extremely heterogenous in its service provision. In general, we found reported benefits of the link worker model of social prescribing. For example, link workers are able to spend a relatively long time with clients and provide targeted support to clients. This is in line with the findings of previous studies.^16^

But our results also revealed multiple challenges to evaluation of the service, and large variation in the different outcomes assessed by current service providers. It seems the fragmented implementation of the link worker model of social prescribing makes evaluating the impact of ‘the service’ extremely challenging. As we have shown in our study, the current service cannot be evaluated nationally a single link worker model.

We identified various routes clients could take through link worker services. Each appeared appropriate but were locally contingent. There appears to be no systematic process to identify and select services for onward referral of clients. Instead, onward referral appears to depend mostly on link workers’ local knowledge and networks. This echoes the findings of the recent National Voices report, which reported that link workers recruited by primary care networks within the NHS have less access to existing community networks and, therefore, find it hard to identify the right sources of support and make referrals.^7^

Not all the variation we identified was appropriate and justified. We found differences in how individual link workers are allocated caseloads and the resources available to them. The provision of additional support (such as training) or of satisfactory reporting structures varied significantly across the country. In some places, link workers were seen as a financial burden on host organisations because link worker overheads were not provided.

Participants emphasised that service funders need to better engage with those who provide social prescription, to understand the complex relationship between link works, clients, and onward referral. A shared vision of social prescribing is essential to develop and foster this closer collaboration. Although some successes were reported in our findings, we uncovered some major professional and organisational boundaries and barriers between link workers, health care professionals, and VSCE workers that must be overcome.

A large challenge to an evaluation of the link worker model is that individual outcome data are not routinely collected and that client outcome measures are not consistent across services. We found no routine data collection on whether people referred to link workers completed the consultation or where they were referred on to for support.

However, we have shown that not all services have access to or use electronic health records. A recent study used Royal College of General Practitioners Research and Surveillance Centre data to examine social prescribing.^17^ This database of more than 500 GP practices across England^18^ was used by researchers to establish social prescribing indicating social prescribing offered, social prescribing referral made, and social prescribing declined. Nevertheless, as identified by our interview participants, referral to a link worker does not equate to appropriate use of the service and does not give any information on the value of the link worker intervention. The generic measures used in various regions (Table 1) offer a valuable opportunity to identify changes in health and wellbeing. However, without systematic reporting or coordination or collation of these wellbeing measures across and between link workers nationally, they will not be useful for evaluation.

Participants were mixed about the ideal outcomes that should be measured to evaluate the link worker social prescribing service. We found evidence to suggest that existing data systems might be co-opted for evaluation, for example, using electronic health records to retrospectively evaluate services.^17^ However, these methods work best for very defined medical conditions. Defining a standard outcome set for all social needs is challenging.^17^

As we have highlighted in the findings, determining the effectiveness, cost-effectiveness, and return on investment of the link worker model of social prescribing as it currently functions, is likely not possible. A common outcomes framework approach for social prescribing has been described by NHS England.^19^ This describes three impacts: on the person (such as health and wellbeing), on community groups (such as changes in number of local volunteers, their capacity to manage referrals), and on health and care systems (such as changes in demand on GP and hospital services).^19^ These three areas will require metrics to determine success, however, our results suggest that there are several limits to the ability of the NHS to develop, collect, and report such metrics.

Finally, social prescribing relies heavily on services provided by community groups, small charities, and VCSEs. Our study identified fundamental issues which may hinder an impact evaluation, most notably the changing capacity of VSCE services and their unstable funding structures. Community and voluntary services may have different organisational goals, and often depend on volunteers, and intermittent (often underfunded) external funding. Despite the growing demand for link workers and community service providers, many participants reported a steady decrease in funding since 2018/19. Social prescribing services will not be sustainable if onward referral services are not equally supported.^19^

### An exploration of evaluation study designs

Using the evidence generated in our feasibility study, we propose three approaches to evaluating the link worker model of social prescribing.

The first is a retrospective matched cohort analysis, based on available routinely collected anonymised health care data. The evaluation would be restricted to specific health conditions and control groups matched for several relevant characteristics. This approach should not introduce additional burden to the current service. Compared to other designs, it would be timely and lower cost, as it would use real-world evidence. However, collecting only clinical outcomes would not reflect the full impact of social prescribing and, therefore, we recommend this approach only for a more focused study. That is, one disease area or clinical indication (e.g. Ways to Wellness, which is for specific long-term health conditions).^20^

The second option is to conduct a pre- and post-observational study, combining quantitative and qualitative study designs. This would require identification of sites that have not yet implemented the link worker model of social prescribing or sites that want to change their approach to service delivery. A pre- and post-observational study would involve and allow for additional data collection. Therefore, relevant outcomes – such as service satisfaction, quality of life, and economic outcomes (e.g. cost-utility, cost-benefit, or cost-effectiveness) – could be consistently collected across different sites at predefined timepoints.^10^ Qualitative data could be collected to capture the client, link worker, and VCSE experiences of services. While this approach would allow for a more in-depth evaluation, it would be resource-intensive and subject to the biases associated with observational study designs (such as selection bias, information bias, and confounding).

The third option is a realist evaluation, which could provide an understanding and explanation of how and why the link worker model does or does not work in specific contexts and settings. In a realist evaluation, researchers would initially explore and identify programme theory/theories using realist interviews, stakeholder consultations, and review of key documentation and data. Findings would generate context-mechanism-outcome configurations, which seek to explain the service. These configurations would then be iteratively tested in various comparative contexts to develop a more informed programme theory. Data collection would use multi-methods, and further testing and refining of the programme theory with stakeholders and community members would be required. We consider this approach would achieve the most comprehensive analysis. It enables in-depth understanding of social prescribing from those directly involved in its delivery and allows researchers to capture the complexity of the service. However, it is resource-intensive and needs to be conducted by researchers trained in realist approaches. Further, the realist evaluation may produce more abstract depictions and lessons of what the link worker model can achieve, rather than a prescriptive road map of how to deliver successful services. It cannot determine effectiveness estimates of the intervention under investigation.

There is a drive towards population health management, an approach which aims to improve health and wellbeing of specific populations using data to guide the planning and delivery of care for the target population and reduce health inequalities across the population.^21^ This could facilitate social prescribing that applies specific approaches for defined target populations (such as people with specific conditions or wider health determinants). This would help to reduce the heterogeneity in the current approaches described above and potentially make evaluation of the link worker model more straightforward. Furthermore, a project evaluating social prescribing link workers is underway.^22^ The evaluation involves various methodological approaches to evaluate link worker services across various parts of the UK.

## Limitations

There are three main limitations to this study. First, interviews were conducted during the COVID-19 lockdown. This reduced our potential sample size (some people were unavailable) and restricted the interviews to virtual formats (MS Teams and telephone). This was not planned in the original protocol, where we had anticipated informal observation of services and site visits. We also recognise that the COVID-19 lockdown impacted on the provision of social prescribing services including access to and uptake of services. Furthermore, we did not return the transcripts of interviews to the participants for comments and/or correction. This was to minimise burden on participants during an already time limited, intense period for health, and social care services. However, to ensure accuracy, a second researcher checked transcripts against recordings of the data.

Second, we did not actively seek data saturation (that is, a situation whereby additional data collection does not provide any new information) as we aimed for breadth of views across the service delivery environment (i.e. service roles) rather than test a specific hypothesis. Nevertheless, we recognise the uncertainty in the literature as to how saturation should be conceptualised, and inconsistencies in its use.^23^

Third, due to variation in the development and implementation of social prescribing across different countries,^2^ the findings of this study might only apply to countries with comparable settings.

## Conclusion

The nature of social prescribing and the current service delivery model poses major challenges for an impact evaluation of the social prescribing link worker model. Current data collection is limited and inconsistent, emphasising the need for the development of robust data systems. The challenges to service delivery need to be addressed at both policy and practice levels before challenges of evaluation can be fully addressed. Consultation with the wider social prescribing community is essential to ensure plans for future impact evaluations are practical, and do not burden members of the workforce, who are already facing significant pressure.

Future studies should include more in-depth mapping of the heterogenous current service pathways to better understand how services can better use evidence to tailor their provision to local populations.

## Supplemental Material

Supplemental Material - What is the best way to evaluate social prescribing? A qualitative feasibility assessment for a national impact evaluation study in England Supplemental Material for What is the best way to evaluate social prescribing? A qualitative feasibility assessment for a national impact evaluation study in England by Abimbola Ayorinde, Amy Grove, Iman Ghosh, Jenny Harlock, Edward Meehan, Natalie Tyldesley-Marshall, Adam Briggs, Aileen Clarke and Lena Al-Khudairy in Journal of Health Services Research & Policy
