# Supplementary material for: What is the best way to evaluate social prescribing? A qualitative feasibility assessment for a national impact evaluation study in England
Source: J Health Serv Res Policy. 2023 Dec 15;29(2):111–21. doi: 10.1177/13558196231212854 (PMC10910745; doi:10.1177/13558196231212854)
Supplement: Supplemental Material - What is the best way to evaluate social prescribing? A qualitative feasibility assessment for a national impact evaluation study in England [file sj-pdf-1-hsr-10.1177_13558196231212854.pdf]

Online supplement

S1: Interview Guide

Thank for taking the time and speaking to us: your expert inputs and perspectives are extremely valuable to help us understand the requirements for a future link worker social prescribing research evaluation. There are no right or wrong answers, the discussion is aimed to allow us to better understand the current shape of the service and what is required for a future evaluation.

Just to confirm:

- Your participation is voluntary and you are free to stop this chat at any time without having to give a reason for doing so.
- This discussion will be looked at by researchers from Warwick University to help us summarise the requirements for a future social prescribing evaluation. It will not be possible to identify you in any reports.
- This is expected to last up to 40 minutes
- We will record the chat with your permission. This will help us summaries the discussions that we have without including any details that might identify you.
- Are you happy to take part in this chat?
- **Just before I start,** I would like to clarify that we are specifically interested in the link worker model but if you are implementing other approaches/models then can you kindly clarify this during our chat

#	Question	Comments	Tick if asked
**1.**	**I would like to start with some opening questions to learn more about your role in Social Prescribing**		
1.1.	Could you briefly describe your role and background?<br><br>***Internal check:***<br><br>a) *National Lead*<br>b) *Regional lead*<br>c) *Site practitioner*		
1.2.	How long have you worked in this role?		
1.3.	What are the areas/region that your role covers across England?		
1.4.	How is social prescribing being implemented (in your region/area/practice?		
1.5.	Can you tell us briefly about what models or approaches to social prescribing are being developed locally?	Following on from point 1.4.	
**2.**	**We are interested particularly in approaches that use link workers/the link worker model,**		
2.1.	Could you please talk me through the (if applicable):<br>a) Overall structure<br>b) Human resources required/involved<br>c) Protocols that are in place<br>d) Organisations involved<br>e) Domains/themes/condition specific that it covers (for instance exercise, green prescription, arts prescriptions…)		
2.2.	**If site practitioner:** how long have the link worker model been running for?		
3.	**Now I would like to discuss the referred person's journey and some related points** (for regional leads, we could ask them to pick (one or however many) social prescribing services that they know a lot about in their region in order to answer these more detailed questions, if they can)		
3.1.	Could you please talk me through the person's journey through the service of link worker social prescribing model (more around the referral process and pathway)		
3.2.	What are the services/community groups that are likely used by people (for instance highest used vs lowest)?		
3.3.	What data link workers have about the clubs/groups/activities available in their area to refer people to? For instance, do they keep a database of local organisations		
3.4.	What about the nature and length of patient follow-up		

	(prompts: how are they followed up, where are they followed up, are they followed up by the link worker, or by their GP practitioner, or whoever referred them to the link worker social prescribing service)		
3.5.	Could you describe the make-up of people taking up social prescribing (how different are they to the overall practice population, availability of social class data)?		
3.6.	Could you describe how non-attendance data is captured and how is this captured at different stages? For instance: referral to link worker, referral to service, turning up to service, completing the prescription (i.e, the course/staying involved for 6 months)		
**4. Now if we can discuss in more detail the outcomes/data that are captured (in relation to the link worker model)**			
4.1.	What specific social prescribing routine data that are captured pre and post referral?<br>(prompt: characteristics of people, list for each condition/service for which people are referred, that someone turns up, that they engage in a service, measures/methods used, and analysis (if any)		
4.2.	Could you describe the methods/resources that are in place to capture the individual's outcomes during this journey?		
4.3.	How often are these outcomes captured (follow-up data across different timepoints)?		
4.4.	How are different data captured, so for instance resulting from different types of social prescribing/community groups including: exercise, green prescription, arts prescriptions….		
4.5.	We have identified some outcomes that are mentioned by the literature, are any of the following routinely collected (if yes, how)?<br><br>Any additional data collection in areas such as Health service:<br>• Prompts: Social care service use, health care use (GP consultations), medications prescribed<br><br>Health and wellbeing:<br>• Prompts: Mental health, Social support, Anxiety or stress, Quality of life, Self esteem<br><br>Physical health:<br>• Prompts: Gym memberships or participation in sports		

	Health management:<br>• Prompts: HbA1c, BMI, systolic blood pressure, cholesterol, smoking<br><br>Service uptake:<br>• Prompts: Compliance or user adherence		
**5. If we can move to costs associated with the link worker model**			
5.1.	Could you discuss the availability of the data on costs associated with recruiting link workers and the implementation of the service? Both to primary care networks and organizations involved		
**6. Before we conclude, just a few final points around enablers and challenges of the link worker model**			
6.1.	Could you talk us through potential strengths and limitations of current service of SP in general ?		
6.2.	What are the major enablers and challenges of developing and implementing the link worker social prescribing model?		
6.3.	If we wanted to know SP was working or not/how well it is working across different sites, what challenges would you anticipate? (prompts, dig deeper, any evaluations available that you are aware of)		
**7. Do you have any conflict of interest to disclose?**			
Could you please suggest someone(s) who might help us further in this research? (in terms of data, snowball sampling etc.)<br><br>Last but not least, do you have any question for me?			

Note: capture feedback on questions that should have been asked

S2: Characteristics of Participants

Role in Organisation	Length of Practice in Social Prescribing	Region
Senior/Lead – 3rd sector organisation	14 years	South West
Senior/Lead – NHSE	20 years	South West
Senior/Lead – Academic and 3rd sector organisation	5 years	London
Senior/Lead -NHSE	at least 6 years	East of England
Senior/Lead -NHSE	2 years	North West
Senior/Lead - NHSE	20 years	London
Link Worker	> 1 year	South West
Link Worker	< 1year	South West
Link Worker	1.5 years	South East
Link Worker	4 months	North East
Link Worker	4 months	South East
Link Worker	5 months	South East
Link Worker	2 years	East of England
Link Worker	11 months	South West
Operations director - NHSE	3 years	National
Regional learning coordinator - NHSE	9 years	North East
Senior/Chief Executive Officer – NHSE and VCSE	5 years	North East
Learning Coordinator - Charity	2 years	North East
Learning Coordinator - NHSE	9 years	Yorkshire and Humber
Manager – VCSE	7 years	West Midlands
Learning coordinator - NHSE and VCSE	10 years	North East
Lead/Learning Coordinator – NHSE and Charity	10 months	North West
Manager - VCSE	6 months	West Midlands
Freelance Director - VCSE	4 years	West Midlands
Program Manager - VCSE	11 years	West Midlands

NHS = National Health Service; NHSE = National Health Service England; VCSE = Voluntary, Community and Social Enterprise

S3: Themes with additional quotations

Theme	Quotes
**Communication and the client journey**	*"So the referral is just a simple task which is just an email, really on the system to the link worker, and that can come from any member of the practice team, it doesn't have to be GP. It can be the nurse, receptionist, admin. And interestingly, in the (?) where we've been longest established we have the highest number of self-referrals, because patients are more aware of that service or are more inclined to contact the link worker direct, which is exactly what we want the bypassing the GP was the not, the GP can't help them with their housing or debt or whatever it may be. And so yeah, it's a very simple straightforward process in terms of the difference.* (Stakeholder, 2 years, North East)<br><br>*"A lot of it's [self-referrals] been word of mouth where you know a neighbour or relative has access to the service and recommended it. And we haven't necessarily had to proactively advertise the service we've never had a shortage of referrals. And because I think if you get a good GP and a good practice, get social prescribing it's a win-win. It happens automatically. and it's just because of the simplicity of the referral process. And we get to steady flow"* (Stakeholder, 2 years, North East)<br><br>*"And also there are drop-in centres or drop-in cafe. So for example in Sheffield. There are several this cold drop-in cafe -essentially*

	*there are places like a cafe where young people can turn up and meet other young people, but at the same time, they've, access to a link worker who can support them, and then help them out and perhaps refer them to other support if needed. So that, their referral sources can be quite a few ." (*Lead, 5 years, London)<br><br>*"Doctors, nurses or anybody can self-refer and comes to her via System One. They fill the referral form and she send a task………. There are self-referral but person must be a patient of the GP service and the process is- self refer to the reception and picked up by the link worker…….."* (Link Worker, 4months, South East)<br><br>*"And we actively encouraged first appointments, and either in the home or in a community setting, unless there are any potential safeguarding issues. And, we, one of the challenges we have within surgeries is lack of space, see patients in the surgery. Well, I guess, in SP point of view The idea is to take people away from health setting to engage them with a community. But I think the feedback we get loud and clear from our team is that you do get a much better understanding of somebody's position, through a visit. And they are much more willing to reach, and share whatever issues or experiences they happen, because once they get what a link worker's role is and that it is a non-medical, you know that the strength of the purpose of the link worker is the relationship and the trust you build up with that patient to understand what the issues that they're going through."* (Stakeholder, 2 years, North East)

	"Our practice is a very deprived area one of the most deprived area of the county and older more affluent people. It tends to be pretty fairly split. We don't have a large number of black and minority ethnic group patients but to be honest we don't have a large black and ethnic minority group. ….. it is not a very diverse population." (Link worker, 4 months, North East)<br><br>*"we've got time to speak to the patients [we] identify things that wouldn't necessarily have come up in a 10 minute GP appointment. …… we're in a really unique position to actually talk to patients and see what matters to them and make a difference to those things that aren't necessarily the medical and clinical"* (Link worker, 2 years, East of England)<br><br>*" The best value is that it will essentially save the NHS funding in the future. Also reduce the demands and dependence, and also reliance on GPs, it's very person centred. So they have a voice, they're empowered to make choices, and it works."* (Link Worker, 11 months, South West)<br><br>*"[SP] has the ability to bring communities together, and grow and develop the voluntary sector if done well, so that communities are thriving and there's much more activity happening on the ground, much more opportunity for people to come together and take part in activities which we know improve health and wellbeing, because we know the opposite of that is isolation and*

	loneliness, which is detrimental to health and wellbeing." (Stakeholder, 10 months, North West)<br><br>*"We have [a SP] steering group which has membership from all 10 localities, and enables us to share best practice. Find out what's going on in different areas. People talk to each other and share information across the 10 localities, which I think is very good."* (Lead, 2 years, North West)<br><br>*"…..it is something for them to think about from service to service depending on what is commissioned and there is in-build 6 weeks review regardless of the trigger episodes / something that changes the pathway or journey, life event or whatever that might be and that for me automatically trigger some sort of review. Whether it doesn't depend on the tracking system"* (Stakeholder, 3 years, National)<br><br>*"Time point vary generally again but more often than not you would have the baseline and three months, then six months stage, and then depending on the intervention or the service, it may go on longer depending on how people what their needs are and so on."* (Lead, 2 years, North West)<br><br>*"In our system elemental we just about to launch in fact our first locality is going to live in about a month or so, and with what they're going to push back into the system……we'll be able to have that push back that will allow a limited amount of data to come*

	back in to the GP system so that the next time they're sitting down with that individual in front of them, they'll be able to see oh! we'll be sending off their SP went to this gardening club. I see that you attended, and the GP will be able to talk that through with the individual that's still in its early stages because what we do need to do is to work out what exactly we're going to refer. That's the anticipation, we'll be able to have that push back." (Lead, 2 years, North West)<br><br>*"Often, [its] not possible to always find out what's happened. And, you know, communications may be difficult, they may be difficult to get hold of that person again……."* (Lead, 20 years, London)<br>*"yeah, I mean obviously we go out to collect the data about onward referrals, so we've got all of that. And, but it's not necessarily all that useful because everybody's different they want different things clearly things like CAB [Citizen Advice Bureau] for money advice is actually up there, there is art Carer support."* (Lead, not clear - at least 6 years, East of England)<br><br>*"I think generally the community group element is not well recorded, I think after they get referred on to a community group that's when the data goes a bit skew-whiff and we're hoping Manchester the elemental help that because that allows community groups to record data on it but currently that's not captured. So, the data the wellbeing outcomes and where they go on to which I do, by the way, and then recorded with the scheme on whatever their, their system is basically, but we need to get better at recording what happens after people get linked into community groups."* (Stakeholder, 10 months, North West)
Capturing metrics and outcomes	*"And then after the case is live with a link worker, and, all schemes will have a case management system or a way of recording cases and planned appointments failed to attend will be recorded. Usually after a number of failed to attend the case is closed. And*

	then that'll be reported back to the referrer, but it was closed because it was no longer in contact or were out of contact and if it's suspicious I worry and then usually that's flagged as some kind of safeguard into the referrer as well." ." (Stakeholder, 10 months, North West)<br><br>*"I don't think we've got particularly good information about that, and that's for a couple of reasons. So first of all, we don't have a lot of non-attendance because we visit people at home, so that would be refusing to open the door, and on the whole our link workers will come back another day and check. Because obviously, can be quite concerning if people won't respond having said they would. There is a certain amount of having to reorganise because people have changed their mind, or can't do it on that particular day something happens but we tend to persevere because we know that some of the people who need to help most are the ones most likely to kind of get cold feet and cancel. In terms of once they start attending groups and stuff really that comes back to what I was saying about policy from the bat. So if it's a carer, and they joined a carers group, and we're assuming that the carers group will keep an eye on them and check what happens to them. If they stopped turning up and stuff so we wouldn't normally be collecting the data on that, I don't think…"* (Lead, not clear - at least 6 years, East of England)<br><br>*"Everything goes to EMIS, if they do not attend it goes to EMIS as did not attend. If someone attended but do not engage, then it will be coded as SP declined."* (Link worker, 4 months, North East)
Major enablers and challenges to development and implementation SP service	*"I think a lot of people don't know what SP is. People in the system understand what it is but for common people 9/10 don't know and thinks it is a type of medication. When they are into the service they feel relax and liked it."* (Link worker, < 1year, South West)

	"I think in terms of challenges. There's a lot to be honest, the way we've got such variety of models is good, some point we still don't know what are the key elements that are really strong in SP models. What are the things that we need to replicate and not only what but how. So what are the key elements of that. So we've got various models from voluntary sector led models primary care led models, different models that focus heavily on health outcomes, we've got ones that don't. We've got factors like self-referral that some schemes are really passionate about some schemes don't recognize that as kind of an important element of social prescribing." (Stakeholder, 10 months, North West)<br><br>*"I think in some areas, a challenge is actually knowing what's out there sometimes we spoke about directories a little bit. But I think that, it's not just knowing where somebody can be referred to, but what the availability of that is, and making sure that there's a good availability across an area so maybe some, more asset mapping might be useful to, to help that side of things. And we've spoken about the digital I think that has been a challenge. Some areas haven't collected the information in a way that's easy to access activity and look at outcomes. I think we're on a journey with that one I think that one's getting better. But it's still, not quite there with it. But I think that that is definitely improving."* (Lead, 2 years, North West)<br><br>*"The challenge is convincing the health service and other referrers of the value and the worth in the community sector and the weight of that, actually going into a singing group is as powerful to health outcomes as medicine. I think it's our chance to really change things and route people's health in communities, and that medicine and structured health is something that fewer people need and rely on. But we need everyone to buy into that and that needs to be the first point of call if we're going to crack this."* (Stakeholder, 10 months, North West)

	"it's a very unfortunate term because people think they're going to be prescribed something they need to tell them something they could do or something they can have. When in fact, it's not that at all it's much more coaching model." (Link worker, 1.5 years, South East)<br><br>*"When you look at NHSE guidelines for what a link worker is expected to do without any training except a three hour online, course from Health Education England its frankly ludicrous. The model tends to talk about coaching and motivational interviewing …. Well, that's all very well and good but people are trained for years to be able to do that. And I certainly haven't had any training on it. I don't think most of my colleagues have either."* (Link worker, 1.5 years, South East)<br><br>*"My view is that Health Education England regionally being commissioned to provide the training packages, leaning on the support of the organisations and the voluntary sector organisations and NALW (National Association of Link Workers) to understand what that training looks like. So there needs to be more collaborative approach between the experts in the sector…. to develop what that package should look like."* (Stakeholder, 3 years, National)<br><br>*"We found that the introduction of NHS-SP destabilised the local market because they were offering salaries far in excess of the current rate locally for that level of job. Most experienced staff have moved to those roles as they are paying higher rates."* *(*Stakeholder, 9 years, North East)

	"…… within our service we maintain a database, which is supported by a wide range of volunteers who contact groups and organizations to check the information that we've got is up to date and relevant. and the information that we collect is- I should write so that we can search based on when somebody is available, the price of activity location of activity, day of the week activity that kind of thing. So we collect information and around making sure what the name of group is, the provider and description of the activity, contact details, location, how much it costs and communication information. And they put relevant policies and procedures in place. If there's a waiting list, capacity particular age limit, and when the activity is provided for any access issues to note, or an availability of access requirements, kind of thing. And, and we make note to them again on a regular basis." (Stakeholder, 7 years, West Midlands)<br><br>*"….There are people talking about creating other very clever IT platforms that will draw their data from any number of directories into a single search function. If all the different directories use common data standards. As one way of trying to overcome this problem. But it's a problem that I have been hearing about for well over a decade. And it's, it's also very difficult- organisations that provide services, often don't update their entries about services so that the information goes out to date." (Lead, 20 years, London)*<br><br>*"……. there's a lot of additional on costs, not quite as straight forward. And we know that the primary care network funding is quite restricted in terms of what that cost can be used for. But there are additional on costs, which means that we've had to find commissioned and funding from other support approach"* (Stakeholder, 7 years, West Midlands)

	"One issue that that came up for our service was the fact that [the funding] only covered salary costs. There was no overhead, there's no admin costs. And that has been an issue for us because we don't have office space, we don't have administrative support. So, yeah, I think that was, an issue for the GP, they were a bit surprised that NHS England was only providing them with a salary and no overheads." (Link worker, 1.5 years, South East)<br><br>*"And we know this through our regional work that there are link workers of the moment who don't have access to a laptop who don't have a phone. We're obviously expected to be in the surgery using equipment there. And because the surgeries aren't doing that stuff at the moment, they've not been able to function as well during COVID as our troops have been a function. So I think the only way we were able to make this work was that the NHSE told us back in May last year that we could use money that wasn't reimbursed to the PCN and it became discretionary money for the CCG [Clinical Commissioning Group] we could then, thereby use it as these fees which we've done. And as a result, we've now got 13 Link workers out of 16 within the voluntary sector. And three directly employed by PCN who aren't part of a strategic approach."* (Lead, not clear - at least 6 years, East of England)<br><br>*"And we felt really strongly that actually if you were going to refer on to a voluntary community sector organisation, then you had to help support that voluntary community sector organization with its costs. I think there was some movement before COVID-19 on agreement that management costs to some of that was starting to get covered, there still was no resolution on how they would support the voluntary community sector……………" (* Stakeholder, 9 years, Yorkshire and Humber)

	"…..but as a small charitable organization. We've had very little resource made available to see for example my cost and the operational lead, which are for me now are crucial now that the team's expanded to have that infrastructure that support link worker…………. I think that would be the time and effort to recruit and induct and get somebody embedded in the surgery is quite intense for a small charity." (Stakeholder, 2 years, North East)<br><br>*"One other challenge I would say is probably clinician's own education. We need to really change, you know, clinicians who go through medical school for Lord knows a decade, are taught a very via medical understanding of health. And if we want clinicians to really fully understand social prescribing. We need to change what they're taught in medical schools. We need to get them to be taught what some of us call a social model of health. And this is very difficult if you spend a decade training somebody to understand, health, or something very biomedical then expect them to unlearn. A large portion of what they've been spent 10 years learning to read learn it, we would do better to start off with the teaching of a social model of health in medical school, but that that is a challenge for social prescribing."* (Lead, 20 years, London)<br><br>*"They [link workers] have team meetings together, they have team training and learning opportunities together, they are able to contact each other. And therefore, they feel supported they're able to grow and develop together. And they're able to help each other you know when they come across a patient who's got a problem that they've never come across before."* (Lead, 20 years, London)

	*"But I think eventually BBB (Bromley by Bow) helps to deliver one of the only accredited training program for link workers, which is a level three qualification. I think we need to massively scale up the delivery of training for link workers. I think it's also is important because they need to be safe. They need to know how to do their job well. They will be dealing with, particularly vulnerable people. They will undoubtedly come across, significant safeguarding issues, but the people they work with. They need to know exactly what to do in those situations. And I think that, you know, training is really important for them [link workers]. I think also it's important in terms of their own professional esteem. They are part of a system, full of very highly qualified people, doctors and nurses and health care systems. And I think it's helpful if social prescribing eventually has a widespread work workforce with this widespread with qualifications, because then that will gain more recognition from the other colleagues within the health system as well. Having said that, I wouldn't want it to be a requirement that all link workers had to have a qualification. I think there are some people who work in our communities who would make absolutely brilliant link worker who may struggle to do a qualification for whatever reason. So I don't think it has to be a requirement but I do think we do need to move towards having a more qualified workforce amongst the workers." (*Lead, 20 years, London)<br><br>*"You need to get the **relationships** right, right within the GP practices, and the primary care networks. …………. you need that relationship with patient. The public or the person that the service user needed the relationship certainly with the local health professionals, whether they are GP and GPs are crucial to it. But you also need in the relationship with the volunteer community sector to manage that. You also needed the **resources**, in terms of money and people. And you need the resources out there in the sector. And then finally, you needed the **research**, in order to demonstrate the work to get social prescribing implemented, but you*

	also needed some research as to what effect it had got because you were asking systems to make very difficult decisions with limited money." (Stakeholder, 9 years, Yorkshire and Humber)
Challenges and enablers of evaluating the SP link worker model	*"….it's measuring the softer things like just feeling a greater sense of belonging, so you can use to how do you effectively measure that. I think that's going to be one of the key challenges because it's so subjective. If you do an outcome measurement with somebody, they might have said the bad news, you might be feeling well that day that measurement is that point in time, it doesn't measure how that person feels, in general, and they may have improved things, but they still feel bad, or feel bad at that point in time. I think it's really hard to measure that holistic picture."* (Stakeholder, 10 months, North West)<br><br>*"…the evaluation that Hallam University did….. It did impact on service reduction, it did reduce any attendances, it did reduce hospital admissions, and it did reduce GP appointments, Whether it could be completely categorically down to social prescribing intervention. It's hard to tell."* (Stakeholder, 9 years, Yorkshire and Humber)<br><br>*"What we know isn't happening, is [to] measure how many people got back into work, how many people have got their benefits sorted out, that's all happening. And, again, that data will be collected by the people who have had the baton passed on to them."* (Lead, at least 6 years, East of England)<br><br>*"…people tend to look at the world in terms of systems. And that's not actually how the world works, we have some link workers*

	[who] are absolutely amazing. And everybody thinks they may have some others who aren't quite so amazing... It's nothing to do with the link worker role itself its to do with how it's done by that particular person. A lot of this is about making sure that the right skills are in the right place at the right time." (Lead, at least 6 years, East of England)<br><br>*"We need to be measuring things like number of community groups, attendance at community groups, resilience of community groups, size of voluntary actions. (*Stakeholder, 10 months, North West)<br><br>*"… Make sure kick off data is sufficiently good and well collected. What link workers are telling us is that they are collecting data and what they see in a report at CCG [Clinical Commissioning Group] are completely different what they thought they told them in the first place. There is a big underestimation of people getting intervention - very important one."* (Lead, 14 years, South West)<br><br>*"I suppose also what you want to capture is not just the numbers. This is one of my big soapboxes so I was trying not to go on a rant about it, but qualitative information in these surveys, is really key. Not just constitute information, because I can see if you want me to see 350 patients a week and give them all the leaflet and say that I've done social prescribing then of course. That looks great, doesn't it from a quantitative point of view, I've got 350 people, throughput a week. What's it actually achieving very little those people will still be coming back to the gym, again and again and again and the problems are not getting sorted out. So this is about quality of service as well the quality of time and investment. And I feel strongly that the tour (patient journey) needs to capture. You know the quality of the work, not just the quantity of the work, because there's a tendency to love number than alphabet*." (Stakeholder, 10 years, North East)
